# Supplementary material for: Creep constitutive modeling of the shear strength of the permafrost-concrete interface considering the stress level at -1°C
Source: PLoS One. 2024 Apr 30;19(4):e0297824. doi: 10.1371/journal.pone.0297824 (PMC11060532; doi:10.1371/journal.pone.0297824)
Supplement: Attachment — Submitted filename: Response to Reviewers.docx [file pone.0297824.s001.docx]

Dear Editors and Reviewers:

Thank you for your letter and for the reviewers comments concerning our manuscript entitled“**Creep constitutive modeling of permafrost-concrete interface shear strength considering stress level**”(ID: **PONE-D-23-35040**). Those comments are all valuable and very helpful for revising and improving our paper, as well as the important guiding significance to our researches. According to your nice suggestions, we have made extensive corrections to our previous draft. We have added necessary data to supplement our results and edited our article extensively. The detailed corrections are listed below.

Responds to the academic editor and reviewer's comments:

Academic editor's requirements:

**1.** Please ensure that your manuscript meets PLOS ONE's style requirements, including those for file naming.

**Response:** Based on the downloaded template files, we have revised the article to ensure meet PLOS ONE's style requirements.

**2.** Please update your submission to use the PLOS LaTeX template.

**Response:** I'm sorry we're not proficient in using LaTeX for paper layout. But we reformatted the content of the paper according to LaTex's formatting requirements

**3.** We suggest you thoroughly copyedit your manuscript for language usage, spelling, and grammar.

**Response:** We apologize for the language usage, grammar and spelling issues in our paper. We have checked and changed the full text.

**4.** We note that the grant information you provided in the ‘Funding Information’ and ‘Financial Disclosure’ sections do not match. When you resubmit, please ensure that you provide the correct grant numbers for the awards you received for your study in the ‘Funding Information’ section.

**Response:** We are very sorry for not matching the grant information in the ‘Funding Information’ and ‘Financial Disclosure’ sections. We identified the grant information as “The research described in this paper was financially supported by the National Natural Science Foundation of China (Grant No. 41902272) and the Basic Research Innovation Group Project of Gansu Province, China (Grant No. 21JR7RA347).”

**5.** Thank you for stating the following in the Acknowledgments Section of your manuscript: "The authors appreciate the National Natural Science Foundation of China (Grant No. 41902272) and Basic Research Innovation Group Project of Gansu Province, China (Grant No. 21JR7RA347)." We note that you have provided funding information that is not currently declared in your Funding Statement. However, funding information should not appear in the Acknowledgments section or other areas of your manuscript. We will only publish funding information present in the Funding Statement section of the online submission form. Please remove any funding-related text from the manuscript and let us know how you would like to update your Funding Statement. Currently, your Funding Statement reads as follows: "The author(s) received no specific funding for this work". Please include your amended statements within your cover letter; we will change the online submission form on your behalf.

**Response:** We are very sorry for adding funding-related text to the manuscript. As required by the journal, we have removed funding-related text in the manuscript. In addition, we identified the Funding Statement as “National Natural Science Foundation of China (Grant No. 41902272) and the Basic Research Innovation Group Project of Gansu Province, China (Grant No. 21JR7RA347).”

**6.** In your Data Availability statement, you have not specified where the minimal data set underlying the results described in your manuscript can be found. PLOS defines a study's minimal data set as the underlying data used to reach the conclusions drawn in the manuscript and any additional data required to replicate the reported study findings in their entirety. All PLOS journals require that the minimal data set be made fully available.

**Response**: We apologize that we did not specify where to find the minimal dataset on which our experimental results are based. We have uploaded the data into an online database, which can be found at the link below.

[https://doi.org/10.6084/m9.figshare.249356**73.v2**](https://doi.org/10.6084/m9.figshare.24935673.v2)

**7.** We note you have included a table to which you do not refer in the text of your manuscript. Please ensure that you refer to Table 3 in your text; if accepted, production will need this reference to link the reader to the Table.

**Response:** We are very sorry that we have omitted the description of Table 3 in the text. We added a description concerning Table 3 in the text.

**8.** Please upload a copy of Supporting Information Figure/Table/etc. Supporting information which you refer to in your text on page 22-23.

**Response:** We are very sorry that we have not uploaded supporting information. We have uploaded a copy of Supporting Information in our text on page 17.

**Reviewer #1:**

1. **Comment:** Title: The title should explicitly convey that the creep tests were conducted under -1°C conditions, a crucial detail absent from the current title. We understand that the terms of hazard, vulnerability and risk are different. That is important clarify this concern by the authors. Please check all text and abstract for this concern.

**Response:** As suggested by the reviewer, the test conditions for creep testing should be indicated in the title. We added the -1°C condition to the title in the paper. We understand the difference of hazard, vulnerability and risk. Hazard is a potential adverse condition, vulnerability is the sensitivity of a system or individual to a hazard, and risk is the result of the interaction of hazard and vulnerability, combining likelihood and potential damage. We have changed the contents of the abstract and text to avoid this problem.

2. **Comment:** Abstract: The abstract requires reorganization to emphasize the primary research findings, adopting a logical structure that briefly covers introduction, problem statement, methodology, results, and conclusion. Including quantitative information in the abstract, such as key results, will enhance the reader's understanding of the study's significance.

**Response:** As suggested by the reviewer, the abstract should briefly cover the content of the research and include quantitative information on key findings to enhance the reader's understanding of the significance of the study. Therefore, the abstract section of the study was rewritten and quantitative information on the main results was added to enhance the reader's understanding of the research.

3. **Comment:** Introduction: The literature review needs completion, and it's unclear what sets this paper apart. Updating the literature review is essential, considering recent works like <https://doi.org/10.1007/s12517-021-09394-0.> The introduction should better articulate the research's importance and contributions.

**Response:** As suggested by the reviewer, the introduction should better articulate the research's importance and contributions. Therefore, we completed the literature review and set forth the difference of this paper, recent works were added in line 71-93 of this paper.

4. **Comment:** Introduction: Provide additional details about the research gap, emphasizing the significance of your study. Ensure a smooth transition from the introduction to the subsequent sections of the paper.

**Response:** As suggested by the reviewer, the details about the research gap and significance of study are necessary, so we have added the detail differences of research to ensure a smooth transition from the introduction to the subsequent sections of the paper The addition is in line 94~117 of this paper.

5. **Comment:** Method: The test details lack clarity, and material properties are missing. A more detailed description of the test methods, including material properties, is necessary for comprehensive understanding.

**Response:** As suggested by the reviewer, we have added experimental apparatus, test methods and material properties in lines 234~304.

**6. Comment:** Results: Provide additional context for the results by discussing their relevance to the research question or hypothesis. Address any limitations and potential sources of bias or error in the study.

**Response:** As suggested by the reviewer, we have added to more information about two hypotheses in the text and explained the limitations of the study and the sources of the bias at the results. Added in line 140-145, 150-155, 353-360

7. **Comment:** Discussion: Expand on the broader implications of the findings and their relevance to the field or real-world applications. Strengthen the interpretation of the results to make the paper more engaging.

**Response:** As suggested by the reviewer, we have illustrated the linkage of this study to practical engineering and explain how the results are complementary to the current study. In line 397-414 of the text.

**Comment:** Conclusion: Enhance the interpretation of results in the conclusion, supporting it with quantitative findings. Provide a more detailed conclusion that thoroughly summarizes the study's outcomes. Incorporating these revisions will improve the clarity, coherence, and overall impact of the paper.

**Response:** As suggested by the reviewer, quantitative and comprehensive conclusions are important to the clarity and coherence of the paper. Thus, we add detailed conclusions and quantitatively describe the main findings to improve the clarity, coherence, and overall impact of the paper.

**Reviewer #2:**

1. **Comment:** Lots of equation parameters are not defined or introduced, please verify.

**Response:** As suggested by the reviewer, we have added some definitions and descriptions of equation parameters.

2. **Comment:** The statistics of related to how well the model fits the experimental data are not presented.

**Response:** As suggested by the reviewer, the statistics are very important .We listed the statistics for the fitted data in Table 1

3. **Comment:** Table 5 should be modified and reaaranged, the presentation is not adequate.

**Response:** As suggested by the reviewer, we have reorganized table 5 to make it clearer and more adequate.

4. **Comment:** Conclusions should be presented using bullet points to clearly highlight each of the findings.
5. **Response:** As suggested by the reviewer, conclusions should be presented using bullet points to clearly highlight each of the findings. We presented the findings of the paper in points, highlighting each research finding.

We tried our best to improve the manuscript and made some changes in the manuscript. These changes will not influence the content and framework of the paper. And here we did not list the changes but marked in red in revised paper.

We appreciate for Editors/Reviewers' warm work earnestly, and hope that the correction will meet with approval.

Once again, thank you very much for your comments and suggestions.
